# "Strong Teeth": an early-phase study to assess the feasibility of an oral health intervention delivered by dental teams to parents of young children

**DOI:** 10.1186/s12903-021-01608-x

**Published:** 2021-05-17

**Authors:** Erin Giles, K. A. Gray-Burrows, A. Bhatti, L. Rutter, J. Purdy, T. Zoltie, S. Pavitt, Z. Marshman, R. West, P. F. Day

**Affiliations:** 1 Department of Paediatric Dentistry, School of Dentistry, University of Leeds, Leeds, LS2 9LU UK; 2 School of Dentistry, University of Sheffield, Sheffield, S10 2TA UK; 3 Bradford Community Dental Service, Bradford District Care NHS Foundation Trust, Bradford, BD18 3LD UK

**Keywords:** Caries, Training, Behaviour change, Paediatric, Prevention, Parents

## Abstract

**Background:**

Tooth decay (caries) is a significant health burden in young children. There is strong evidence for the benefits of establishing appropriate home-based oral health behaviours in early childhood. Dental teams are well placed to provide this information and there is clear advice on what oral health information should be given to parents. However, research has shown that there is limited guidance, training and resources on *how* dental teams should deliver this advice. "Strong Teeth" is a complex oral health intervention, using evidence-based resources and training underpinned by behaviour change psychology, to support behaviour change conversations in dental practice. This early phase evaluation aims to assess the feasibility of this intervention, prior to a full-scale trial.

**Methods:**

The study recruited 15 parents of children aged 0–2-years-old and 21 parents of children aged 3–5 years old, from five NHS dental practices across West Yorkshire. Participant demographics, self-reported brushing behaviours, dietary habits, a dental examination and three objective measures of toothbrushing were collected in a home-setting at baseline, then at 2-weeks and 2-months post-intervention. Recruitment, retention and intervention delivery were analysed as key process outcomes. Brushing habits were compared to national toothbrushing guidelines – the Delivering Better Oral Health toolkit (Public Health England).

**Results:**

Strong Teeth was feasible to deliver in a General Dental Practice setting in 94% of cases. Feasibility of recruitment (37%) exceeded progression criterion, however retention of participants (75%) was below the progression criterion for the 0–2 age group. More than half of children recruited aged 3–5-years had caries experience (52%). Total compliance to toothbrushing guidance at baseline was low (28%) and increased after the intervention (52%), an improvement that was statistically significant. Dietary habits remained largely unchanged. Plaque scores significantly decreased in the 3–5-year-olds and toothbrushing duration increased in all age groups.

**Conclusion:**

"Strong Teeth" intervention delivery and data collection in the home setting was feasible. There was a positive indication of impact on reported toothbrushing behaviours. Some amendments to study design, particularly relating to the inclusion of the 0–2-year-old group, should be considered before progression to a full trial.

*Trial registration* ISRCTN Register: ISRCTN10709150. Registered retrospectively 24/7/2019.

**Supplementary Information:**

The online version contains supplementary material available at 10.1186/s12903-021-01608-x.

## Background

Oral health is integral to holistic wellbeing [1]. However, dental decay (caries) is one the most prevalent non-communicable childhood diseases in the world [2]. Nationally, by the age of five, nearly a quarter of children in England have experienced caries with an average of three teeth affected [3]. Significant regional inequalities exist between children with and without caries, with deprivation a key risk factor [4]. West Yorkshire has a population of 2.2 million and some of its urban areas are among the most deprived in England [5]. In Bradford, a city in northern England, 36% of five-year-olds have caries experience, significantly higher than the national average [3].

The consequences of untreated caries are well documented and include pain, infection, disturbed sleep and time missed from school [6–8]. Caries creates a substantial financial burden for the National Health Service (NHS), with over £50 million pounds spent annually on tooth extractions in hospital and is the most common reason for a child to have a general anaesthetic [9, 10]. Caries is an almost entirely preventable disease, therefore facilitating parents to introduce positive oral health behaviours during children’s early years, particularly in at risk groups, is crucial to long-lasting good oral health [11]. These behaviours include advice on brushing twice a day with the correct fluoride concentration toothpaste and limiting sugary foods and drinks.

Research by Tickle et al. [12] demonstrated that despite regular attendance at the dentist and receiving oral health advice following national guidance [13], 40% of children still developed caries by the age of six. Although PHE outlines what oral health advice should be given in the Delivering Better Oral Health (DBOH) toolkit [13], it does not suggest how this information should be delivered. Numerous barriers to behaviour change conversations have been identified, including parental attitudes towards oral health, parental motivation and dental professionals’ knowledge, time and resources [14–20]. National Institute of Clinical Excellence (NICE) states that health conversations underpinned by behaviour change theory are more likely to be successful [21]. A recent randomised control trial (RCT) [22] has shown a reduction in subsequent caries in children who have had a dental general anaesthetic, following a behaviour change conversation with a dental nurse trained in motivational interviewing. Encouraging parents to change oral health behaviours for their children requires more than mere information delivery; and the wider dental team need to be supported with appropriate resources and training to undertake this successfully. Few interventions currently exist to tackle this issue.

"Strong Teeth" [49] is a complex oral health intervention underpinned by behaviour change psychology [23, 24] and national guidelines for oral health, the DBOH toolkit [13]. "Strong Teeth" provides evidence-based resources to support the delivery oral health advice and training for dental teams in effective behaviour change conversations. Table 1 outlines the rationale and key components of the intervention. Early-phase feasibility studies are a vital developmental milestone in the intervention development process, exploring the invention acceptability, recruitment and retention of participants, initial signals of impact and anticipating problems prior to a full-scale trial [25]. This paper focusses on the quantitative findings of this early-phase evaluation – qualitative results are reported in a separate paper [26].

**Table 1:** Intervention development using TIDieR checklist [48]

Brief name of intervention	"Strong Teeth" – a complex intervention delivered in general dental practice to the parents of young children
Why (rationale, theory, goal)	Caries is a significant health burden in young children. There is strong evidence for the benefits of establishing appropriate home-based oral health behaviours in early childhood and these benefits persist over the life course. These behaviours include brushing twice a day with fluoride toothpaste and limiting sugary foods and drinks. However, how dental teams effectively support parents to establish these oral health behaviours is uncertain. "Strong Teeth" is a complex oral health intervention, underpinned by behaviour change psychology to support oral health conversations in practice and training in effective behaviour change conversations
What 1. Materials for intervention and training (access to materials) 2. Procedures (describe activities and support activities)	All dental team members delivering the intervention attended a one-day training course, covering evidence-based techniques for undertaking effective behaviour change conversations and guidance on how to use the "Strong Teeth" resources. The "Strong Teeth" resource ‘pack’ was issued to each dental practice, including an oral health chat sheet, conversational flowchart, motivational laminate, posters and four advice leaflets for toothbrushing, healthy eating, behaviour management and advice for friends and family Parent’s choose an area of their child’s oral health behaviours they want to discuss. The parent’s level of motivation is identified and following the flowchart, the professional then undertakes a supportive behaviour change conversation. Parents explore current barriers to this behaviour and encouraged to identify their own simple and achievable solutions. The 3–5-year-olds were given an Oral B electric toothbrush and parents are shown how to use it. A tailor-made action-plan is developed and agreed upon, to be followed-up at the next appointment. Ongoing support and advice were given to the dental practices via telephone and through regular practice visits by the team liaison
Who provided (describe expertise, background, specific training)	The prevention is designed for delivery by Dental Care Professionals – dentists, dental care professionals and dental nurses with additional training
How (modes of delivery, e.g. face to face/individual group)	Face-to-face appointment with the dental professional and parent/child
Where (types of locations)	In dental practice
When and how much (how often is intervention delivered, duration)	For purposes of pilot study, the "Strong Teeth" intervention is delivered in a one-off visit, followed-up over 2 months. However, the intervention has been designed to focus on cyclical, continuous improvement, which could be delivered continually throughout childhood
Tailoring (how will intervention be individualized)	The oral health conversation is guided by parents self-identified barriers to oral health, and an individualised action-plan is created
Modifications (any changes during the study)	Reported throughout manuscript
How well 1. Intervention fidelity assessed by 2. Actual adherence	Recruitment, retention and feasibility of delivery Suggestion of impact in the self-reported and observed measures of parental-supervised toothbrushing
Brief name of intervention	"Strong Teeth" – a complex intervention delivered in general dental practice to the parents of young children
Why (rationale, theory, goal)	Caries is a significant health burden in young children. There is strong evidence for the benefits of establishing appropriate home-based oral health behaviours in early childhood and these benefits persist over the life course. These behaviours include brushing twice a day with fluoride toothpaste and limiting sugary foods and drinks. However, how dental teams effectively support parents to establish these oral health behaviours is uncertain. "Strong Teeth" is a complex oral health intervention, underpinned by behaviour change psychology to support oral health conversations in practice and training in effective behaviour change conversations
What 1. Materials for intervention and training (access to materials) 2. Procedures (describe activities and support activities)	All dental team members delivering the intervention attended a one-day training course, covering evidence-based techniques for undertaking effective behaviour change conversations and guidance on how to use the "Strong Teeth" resources. "Strong Teeth" resource ‘pack’ was issued to each dental practice, including a conversational flowchart, motivational laminate, posters and four advice leaflets for toothbrushing, healthy eating, behaviour management and advice for friends and family Parent’s choose an area of their child’s oral health behaviours they want to talk about. Parent’s level of motivation is identified and following the flowchart, the professional then undertakes a supportive behaviour change conversation. Parents are allowed to explore the current barriers to this behaviour and encouraged to identify their own simple and achievable solutions. A tailor-made action-plan is made, to be followed-up at the next appointment. Ongoing support and advice were given to the dental practices via telephone and through regular practice visits by the team liaison
Who provided (describe expertise, background, specific training)	The prevention is designed for delivery by Dental Care Professionals – dentists, dental care professionals and dental nurses with additional trainings
How (modes of delivery, e.g. face to face/individual group)	Face-to-face appointment with the dental professional and parent/child
Where (types of locations)	In dental practice
When and how much (how often is intervention delivered, duration)	For purposes of pilot study, the "Strong Teeth" intervention is delivered in a one-off visit, followed-up over 2 months. However, the intervention can be delivered at regular dental reviews, throughout childhood, to encourage continuous improvement and maintenance of any positive behaviour change
Tailoring (how will intervention be individualized)	Conversation is guided by parents self-identified barriers to oral health. Individualised action-plan created
Modifications (any changes during the study)	Reported throughout manuscript
How well 1. Intervention fidelity assessed by 2. Actual adherence	Recruitment, retention and feasibility of delivery Suggestion of impact in the self-reported and observed measures of parental-supervised toothbrushing

## Aims and objectives

As stated in the "Strong Teeth" protocol paper [24] the primary aim of this early-phase study was to:

Explore the feasibility of delivering the "Strong Teeth" intervention to parents of children aged 0–5 years old, reviewing study findings against the progression criteria in Table 2:

**Table 2:** Progression criteria to definitive trial [24]

Adoption and maintenance of appropriate oral health bahaviours at 2–3 month follow-up (80%) based on self-report measures
Intervention mechanism produces intended changes in the determinants of oral health behaviour
*Process evaluation*
a. Feasibility of delivering the "Strong Teeth" intervention in a dental setting
b. Intervention, and self-reported and objective outcome measures are acceptable to dental teams and parents
c. Adequate recruitment (25%) of eligible families for data collection
d. Adequate retention (85%) of consented families to data completion

Additional secondary objectives [24] included:

1. To study the mechanisms of action for the "Strong Teeth" intervention

2. To describe the changes in dietary behaviour and parental-supervised brushing (PSB) as a result of the "Strong Teeth" intervention in children aged 0–5 years old

3. To examine the impact of providing children aged 3–5 years old with an Oral-B electric rechargeable toothbrush, with respect to acceptability and tooth- brushing behaviours

## Methods

### Study design

This mixed-methods early-phase feasibility study followed the Medical Research Council (MRC) framework for ‘Developing and evaluating complex interventions’ [25].

### Participants

Best practice recommendations [27] advise pilot studies to use a sample of at least 30–providing a 95% confidence interval (CI) of no more than ± 17.9%. We aimed to recruit 40 parents and children (dyads): 20 parents of children aged 0–2 years old and 20 parents of children aged 3–5 years old. Anticipating a 15% loss to follow-up, the achieved sample size of n = 36 was sufficient.

Using professional contacts, dental practices working in West Yorkshire were approached to participate in the study and deliver the "Strong Teeth" intervention. Each dental practice was asked to identify a range of participants who were representative of the local population. For inclusion—parents had to be present during the home visit, needed to speak English and have no other children already participating in the study. The practices contacted suitable dyads due for their routine dental examination by telephone and briefly explained the study. For those who showed an interest in participating, study information was sent by post and contact details were shared with the research team. If willing to take part, a baseline home visit was organised to undertake consenting and baseline data collection.

### Intervention

A detailed summary of the "Strong Teeth" intervention is outlined in Table 1.

### Data collection

Data collection was carried out over four visits by four dental professionals experienced in paediatric dentistry, supported by three research assistants. For the purpose of this early-phase study, the use of intra oral photographs as a means of calibration was adopted. An experienced British Association of the Study of Community Dentistry (BASCD) examiner provided training to all the dental researchers to ensure a consistent approach to inspection procedures, tooth codes and diagnostic criteria. Dental researchers underwent calibration for caries and plaque detection and their agreement was assessed using Fleiss’ kappa.

Data were collected in the home setting, as the intervention is intended to influence home-based behaviours. Children were examined supine, using a disposable dental mirror and a head torch for illumination. Dental probes were not utilised and cotton rolls could be used to remove debris.

1. First visit – consent and baseline (BL)
  A self-reported questionnaire, a dental examination and three objective measures of PSB: child’s plaque levels per sextant, duration of toothbrushing and frequency of toothbrushing (the specific details of these measures is outlined below).
2. Second Visit – two-week follow-up (2WFU)
  Two weeks following the "Strong Teeth" intervention, further self-reported questionnaire data, a dental examination and three objective measures of PSB were taken.
3. Third visit – Two-month follow-up (2MFU)
  Two months following the "Strong Teeth" intervention, further self-reported questionnaire data, a dental examination and three objective measures of PSB were taken
4. Dyads who completed the study were invited to participate in the qualitative evaluation of the study, via a semi –structured interview [26].

A £10 Love2Shop voucher was issued after each home-based data collection visit.

The self- report questionnaire collected information on participant sociodemographic data, as well as toothbrushing and dietary behaviours, based on validated measures [28, 29]. Self-report determinants of toothbrushing were measured against national guidance. The DBOH toolkit [13] outlines five key items for toothbrushing: parental supervision, strength of toothpaste, amount of toothpaste, frequency and after-brushing habits. A compound measure of ‘total’ compliance to DBOH guidelines was also calculated (i.e. dyads compliant with all items of DBOH advice). Dietary data was collected based on child’s frequency of consumption, using an established dietary questionnaire used within a similar population [28]. Frequency scoring of food and drinks was non-linear (e.g. 0 = none, 1 = less than once a month, etc.).

Teeth were examined for cavitated dentinal caries and restorations, using the BASCD criteria [30]. A dmft score was calculated and child’s plaque levels per sextant were established using the Oral Hygiene Index [31]. Data on frequency of brushing was collected via the ‘Disney Magic Timer’ smart phone application. If dyads did not have access to smart phone, a paper diary to record frequency was issued as a further means of self-reported data collection.

Videotaping of child-parent toothbrushing was undertaken by a member of the research team using a small action camera (GoPro HERO 5, Go Pro Inc). The video was used to evaluate the duration of toothbrushing and scored using methodology described in previous research [32]. Videos also provided an objective method of assessment for the DBOH guidelines and dyad interaction, which is beyond the scope of this article and will be reported in a different paper.

### Data analysis

Reporting continuous outcomes at different time points (baseline, 2 weeks, 2 months), CI’s were calculated using a t-distribution. For categorical outcomes, the CI’s for rate were calculated based on the binomial distribution.

In respect to self-reported toothbrushing compliance to DBOH guidance, repeated measurements were fitted to a multi-level model with timepoints nested within dyads. Maximum likelihood fitting was used rather than reduced maximum likelihood. Within the model, a term was added for ‘time points’ increasing the degrees of freedom by two. This enabled the reporting of compliance rates at each time point and formal statistical testing of the effect of the intervention over time.

The statistical significance of the time term was then determined by use of the log-likelihood ratio test. Similarly, a two-level linear regression was fitted for plaque scores and changes over time tested through a log-likelihood test. This analysis was valid under the assumption that drop out was at random. The analysis was undertaken in the R statistical software environment [33] and using the lme4 package [34].

## Results

The progression criteria to full trial is outlined in Table 2. This quantitative analysis focuses on feasibility and looks to assess process outcomes A, C and D. These relate to feasibility of undertaking the "Strong Teeth" intervention, adequate recruitment (> 25%) and adequate retention (> 85%) of participants.

### Inter-examiner reliability

Inter examiner reliability of scoring from inspections was assessed using Fleiss’ kappa, where a value of 0.85 showed that scores were very highly significantly different from results that would have been obtained at random (p < 0.001), showing a high level of agreement.

### Recruitment and retention

**Fig. 1:**
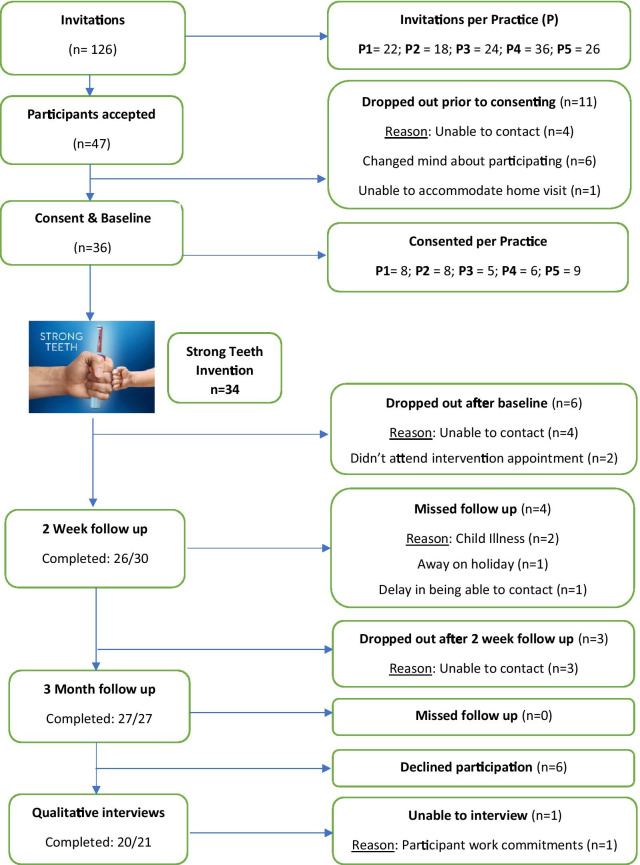
Participant flowchart, as outlined by CONSORT [47]

Five (n = 5) NHS dental practices (with postcodes across Leeds and Bradford: LS10, BD2, BD4, BD6 and BD14) were recruited to deliver the "Strong Teeth" intervention. Dyads were recruited over a 6-month period between October 2018 and April 2019 (Fig. 1 shows the recruitment flowchart). From 126 invitations, 36 dyads were recruited—21 aged 3–5 years-old and 15 aged 0–2 years-old, resulting in a recruitment rate of 37%. Nine participants were lost over the course of the trial, with the large drop-out occurring in the 0–2-year-old group (n = 6), resulting in a retention rate of 75%.

### Demographics

Half (n = 18) of dyads lived in the 10% most deprived areas of England, according to the Index of Multiple Deprivation (IMD) 2019 [5]. At baseline, no children in the 0–2-year-old group (n = 15) had active caries or caries experience; however, 52% (n = 21) of 3–5-year-olds had caries experience, with nine children having active or untreated caries. The mean dmft of the children with caries was 7.9 (± SD = 4.7). Table 3 outlines the baseline demographic data in detail:

**Table 3:** Baseline demographic data

Characteristic	(N = 36)
*Age of parent*	
Mean	35
Range (min, max)	24, 50
% over 30	80.6%
*Birthplace*	
England	86%
Pakistan	3%
India	3%
Ireland	3%
Other	6%
*Number of children*	
Multi-child house	83%
*Qualifications*	
None	6%
5 or more GCSEs	8%
A-level or equivalent	28%
University degree	31%
*Employment*	
Currently employed	67%
*Household income*	
Less than £16,100	28%
£16,100 to £ 21,249	11%
£21,250—£27,999	8%
£28,000—£38,399	22%
£38,399 +	17%
Not answered	14%
*Finances*	
‘Living Comfortably’	22%
‘Doing Alright’	56%
‘Just Getting By’	19%
Not answered	3%
*IMD centile*	
Most deprived 1th	50%
2nd	6%
3rd	8%
4th	11%
5th	3%
6th	14%
7th	6%
8th	3%
9th	0%
10^th^ Least deprived	0%

No significant differences were identified in baseline demographic data for dyads who did and did not complete study follow-up, as shown in in Table 4. Therefore, an assumption of random dropout was made for statistical analysis.

**Table 4:** Baseline demographic data stratified by complete follow-up

Baseline	Complete follow-up*		
	No	Yes	p test
Parent age (mean (SD))	35.67 (6.24)	34.57 (6.02)	0.600
Job group %			0.085
1. Modern Professional	6.7	38.1	
2. Clerical and Intermediate Positions	46.7	19.0	
3. Senior Managers or Administrators	13.3	4.8	
5. Semi-routine manual and service occupations	0.0	9.5	
6. Routine manual and service occupations	13.3	14.3	
9. Other	20.0	4.8	
N/A – i.e. never worked	0.0	9.5	
dmft (mean (SD))	2.00 (4.49)	2.71 (4.54)	0.643
Decayed Teeth (mean (SD))	1.60 (4.12)	1.43 (3.06)	0.887
Plaque % (mean (SD))	33.4 (0.17)	37.0 (0.21)	0.858

^*^To ensure a comprehensive comparison of dyads, 'complete' follow-up was defined as dyads who had participated in all three follow-up visits and for which complete plaque scores and full data sets were available (n = 21)

### Feasibility of data collection and intervention delivery

It was feasible to collect data in the home setting, with 89 out of a possible 93 home visits (96%) undertaken by the research team—Fig. 1 summarises the reasons for missed visits. Of the dyads who underwent initial data collection, 94% returned to their registered dental practices for the "Strong Teeth" intervention. Extensive evaluation looking at the acceptability of the intervention to participants and practices is described in the parallel qualitative paper [26]. The average (mean) time between “Strong Teeth” intervention and final data collection visit was 70 days (S.D. 11.2).

### Intervention outcomes

#### Self-reported toothbrushing behaviours

**Fig. 2:**
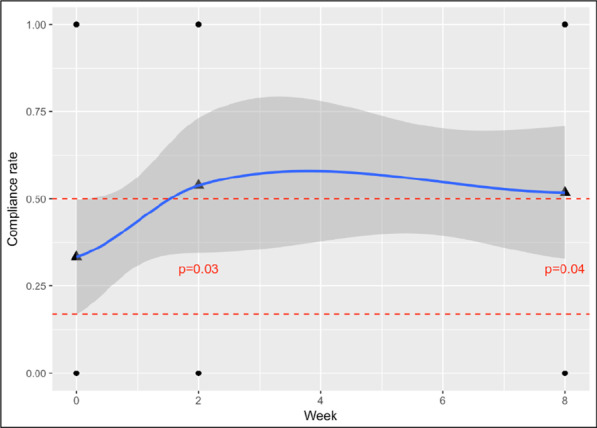
Compliance to DBOH guidance at 2 weeks and 2 months post "Strong Teeth" intervention compliance to DBOH was either 0 (non-compliant) or 1 (compliant for each dyad at each timepoint. Triangles represent the compliance rate at each time point (0, 2, and 8 weeks). To aid interpretation, a smooth (blue line) has been added with confidence envelope shown in darker grey. Horizontal dashed lines show the confidence interval for the baseline rate extended across time. The two later rates are seen outside of this interval

For self-reported toothbrushing habits, ‘total’ compliance to DBOH guidelines increased substantially from 28% (n = 36) at baseline to 52% (n = 27) at two-months following the "Strong Teeth" intervention, and the difference was found to be statistically significant (95%CI = 0.13, 0.42), as represented by Fig. 2. Compliance to all individual components of the DBOH guidelines showed small, albeit insignificant, improvements after the intervention and are shown in Table 5.

**Table 5:** Self-reported toothbrushing pre and post intervention with effect estimate (95% CI)

‘Delivering Better Oral Health’ (DBOH) guidelines	Baseline (n = 36)	Two-week follow-up (n = 26)	Two-month follow-up (n = 27)
Using > 1000 ppm fluoride toothpaste (> 1350pppm for high risk caries children)	88.9% (0.79, 0.90)	100% (1.00,1.00)	100% (1.00,1.00)
Using smear (0–2 years) or pea-sized (3–5 years) amount of toothpaste	75.0% (0.61, 0.89)	76.9% (0.61, 0.93)	77.7% (0.62, 0.94)
Parental supervision	86.1% (0.75, 0.97)	96.2% (0.89, 1.00)	88.9% (0.77, 1.00)
No rinsing after brushing	80.6% (0.68, 0.94)	84.6% (0.71, 0.99)	88.9% (0.77, 1.00)
Spit/wipe toothpaste residue, no swallowing	75.0% (0.61, 0.89)	80.8% (0.76, 0.96)	81.5% (0.67, 0.97)
Brushing at least twice a day, including at night	80.6% (0.68, 0.94)	80.8% (0.66, 0.96)	81.5% (0.67, 0.97)
Total compliance to all DBOH guidelines	27.8% (0.13, 0.42)	53.8% (0.35, 0.73)	51.9% (0.34, 0.70)

#### Dietary habits

There were no significant changes in either age group’s diet after the intervention, however there was a small increase in consumption of certain snacks: cakes (BL: 1.8, 2MFU: 3.4), biscuits (BL: 3.7, 2MFU: 4.2) and sweets (BL: 3.3, 2MFU: 3.8) in the 0–2 age group. Table 6 provides the dietary data for both age groups over the course of the study.

**Table 6:** Dietary data

	Mean frequency 0–2 years		
	Baseline	2 week Follow-up	2 month Follow-up
Water	7.5	7.2	7.8
Milk	6.6	6.8	6.8
Sugared drinks	0.7	0.5	0.8
Cakes	1.8	2.8	3.4
Biscuits	3.7	4	4.2
Sweets	3.3	3.5	3.8
Fresh Fruit	6.4	6.6	7
Vegetables	5.5	5.8	5.8

	Mean frequency 3–5 years		
	Baseline	2 week Follow-up	2 month Follow-up
Water	7.6	7.7	7.6
Milk	6	6.3	6.2
Sugared drinks	1.4	1.6	2
Cakes	3.1	2.9	2.7
Biscuits	4	3.6	4.1
Sweets	4.9	4.4	4.6
Fresh Fruit	6.6	7	6.4
Vegetables	5.8	5.8	5.5

Frequency of food and drinks averaged for all participants across the 3 data collection visits, coded using the following key: 0—None; 1—less than once a month; 2—1–3 time per month; 3 – once per week; 4 – 2–4 times per week; 5 – 5–6 times per week; 6 once per day; 7 – 2–3 times per day; 8 – 4–5 times per day; 9 – 6 or more times a day

#### Plaque scores

**Fig. 3:**
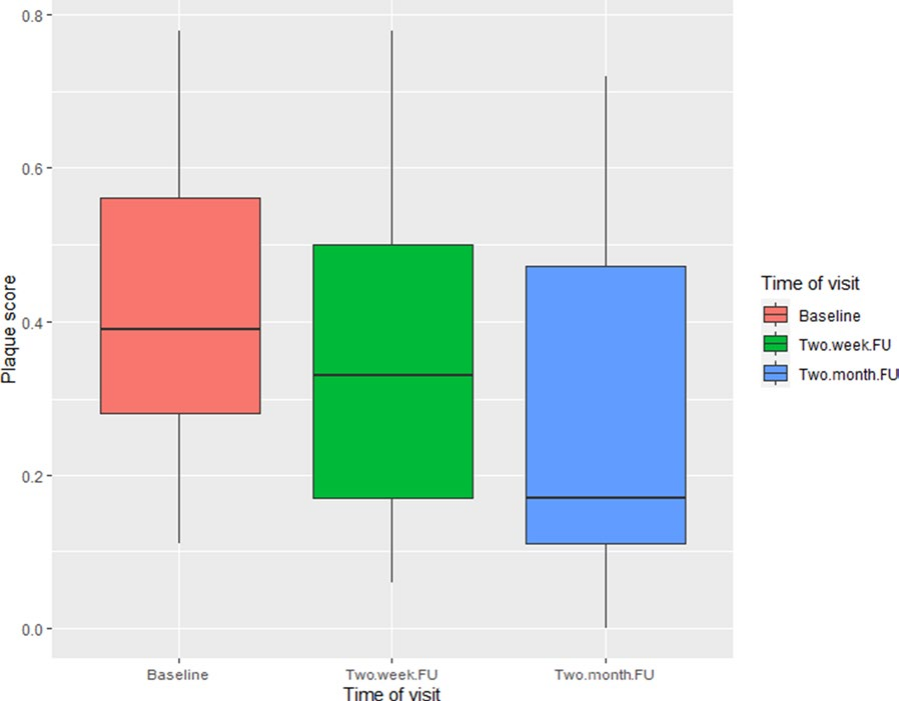
Box plot for plaque scores within the 3–5 age group

Plaque scores were a supplementary measure of objective brushing. In the 0–2-year-old group, plaque scores varied substantially between visits and showed little overall change between baseline (27%, n = 14; 95%CI = 0.17, 0.37) and the final follow-up (26%, n = 8; 95%CI = 0.11, 0.41). There was an incremental decrease in plaque scores in the 3–5-year-olds, the group who used the Oral-B electric toothbrush, between baseline (43%, n = 21; 95%CI = 0.35, 0.51), two-week follow-up (36%, n = 17; 95%CI = 0.25, 0.47) and the two month follow-up (28%, n = 18; 95%CI = 0.18, 0.39). The distribution of plaque scores over time is represented by Fig. 3. The log-likelihood test gave a chi-square value of 6.66 with 2 degrees of freedom, p = 0.036, demonstrating a statistically significant effect of the intervention over time.

#### Toothbrushing duration

Out of 89 home visits, video recording of toothbrushing was available for 98% of participants. Mean toothbrushing duration increased significantly all groups after the intervention, from baseline (76.21 s, n = 35; 95%CI = 65.5, 86.9) to two-week follow-up (88.88 secs, n = 25; 95%CI = 79.8, 98.0) and two-month follow-up (94.20 secs, n = 27; 95%CI = 85.5, 100). There was an increase in parent toothbrushing duration (i.e., parents actively brushing their child’s teeth) from baseline (44.35 secs, n = 35; 95% CI: 29.9, 58.8), which was statistically significant at the two-month follow-up (61.32, n = 27; 95%CI = 49.8, 72.8).

#### Harms

There were no adverse events, serious adverse events or unexpected serious adverse reactions reported during the early-phase study.

## Discussion

The main aim of this early-phase feasibility study was to explore the feasibility of delivering the "Strong Teeth" intervention to parents of children aged 0–5 years old, its impact on oral health behaviours and review study findings against progression criteria (Table 2).

### Recruitment and retention

Adequate recruitment of patients was a key outcome measure, with a target set of 25% of eligible participants. Positively, the early-phase study had a final recruitment rate of 37%. The recruitment of dyads in the 0–2-year-old group was more challenging, however, and took longer than the 3–5-year-old group. In 2019, only 14% of 0–2-year-olds attended the dentist in the District of Bradford [35] reflecting low national rates of attendance of young children in general dental practice [36]. Parental beliefs, such as not needing to take children to the dentist until they have ‘a full set of teeth’ or have a dental problem, have been cited as barriers to child dental registration [37]. Eligibility criteria may have also narrowed potential participants—some parents were unwilling to accommodate a home visit and, due to the lack of access to interpreting services, only families that spoke English could be included in the study.

The study’s final retention rate was 75%, lower than the target set of 85%. Six participants were lost after baseline, which was the biggest single drop during the trial (n = 9), two-thirds of which occurred in the 0–2-year-olds. In this younger, pre-cooperative, age group, examinations were more challenging, as were organising home visits around set routines—potentially barriers for continued participation in the early-phase study. There were only small differences between the 2-week and 2-month visit results – changes were established and maintained at the second follow-up. Options to enhance retention could include reducing the number of follow-up visits or focusing on the older 3–5-year-old cohort.

### Sample

The sample of families in this early-phase study ranged in terms of background, qualifications and income, but demographics were largely representative of the high level of deprivation in the local area [5]. The average age of a parent in the study was 35, which is older than the average age of a first-time parent in England (28.9 years) [39] but reflects a high proportion of study children in multi-child households. None of the 0–2-year-olds exhibited evidence of caries, however, more than half of the children aged 3–5-years-old had caries experience. This prevalence is higher than the local average (36%) for 5 years old children [3]. Critically, the study recruited dyads at high risk of caries based on their demographic backgrounds and the children’s caries experience.

### Feasibility of delivery

Importantly, intervention delivery in dental practice (n = 34/36; 94%) and data collection in the home setting was feasible. The intervention targeted home-based behaviours, therefore collecting data in this environment provides greater insights into home behaviours, as opposed to collecting data in a clinical setting, for example, the ability to film home toothbrushing. Contacting parents and organising visits within a particular time frame did pose challenges for the research team, however the vast majority of data collection visits could be undertaken (n = 89/93; 96%). Data collection in a home setting may have discouraged participation for some, but this did not impact on anticipated recruitment rates.

The measurement schedule was devised using current behavioural change evidence. A recent study demonstrated that the average time for a developed habit to reach automaticity was 66 days [38]. The average time between “Strong Teeth” intervention and final data collection visit exceeded this at 70 days. A longer follow-up period would have increased burden for participants and may not have demonstrated any additional or measurable changes.

### Intervention outcomes

#### Self-reported toothbrushing behaviours

As outlined by Table 2, efficacy was not a primary outcome of this pilot study. Moreover, any suggestion of impact needs to be considered with caution within these small study numbers. Nonetheless, we can report encouraging signs of improvement in oral health behaviours after the intervention. At the final data collection visit, there was a statistically significant increase in ‘total’ DBOH compliance. There were small increases in compliance to individual DBOH items (Table 5), however, these were non-significant within themselves. These findings suggest there was greater behaviour change in the same dyads, as opposed to global improvements within a specific behaviour.

‘Total’ compliance was 27.8% at baseline—much lower than previously reported figures [29, 40, 41]. Traditionally only two or three measures of toothbrushing behaviours have been considered, and this was the basis of the progression criteria for 80% of participants adopting oral health behaviours post intervention (Table 2). A key finding from this study is the low level of compliance when the five-point DBOH criteria is used. This is important, as the absence of any of these five behaviours is associated with dental caries and hence the inclusion in the guidance [13].

#### Dietary habits

There was little reported change in dyad dietary habits over the course of the study. As the intervention is a parent-led conversation based on self-identified oral health barriers, the discussion may have focused more on brushing or a different concern. The efficacy and evidence base for one-to-one dietary interventions at reducing sugar intake is also limited in this age group [42]. There was an increase in sugary food consumption in the 0–2 group, however, this is unsurprising in the context of child development, the weaning process (and the increase in consumption of all types of foods), and is similar to findings of other similar studies [43, 44].

#### Plaque scores

Plaque scores for the 3–5-year-old group (the children using an Oral B electric toothbrush) showed an incremental and significant decrease between baseline visit and follow-up visits. Plaque scores in the 0–2-year olds varied substantially and were more difficult to undertake, which is unsurprising given the behavioural management challenges in this younger age group. This is consistent with other interventional studies that have found a bigger improvement in plaque scores in older children [45].

#### Toothbrushing duration

Active toothbrushing duration showed a positive and significant increase after the "Strong Teeth" intervention, increasing by an average of 18 s. Moreover, active parental brushing increased significantly, by an average of 17 s, also demonstrating an increase in the ratio of parent-to-child brushing. Other studies [32] have showed much shorter active toothbrushing durations in a similar aged cohort, however this was with the use of self-recorded home videos, in the absence of a research team presence.

## Limitations

This early-phase feasibility study recruited participants from general dental practice. Participants recruited may have been more dentally motivated and oral health aware than the general population, although this was not supported by the self-reported behaviours or high levels of caries experience, nor by other RCTs looking at dental attending populations [12]. As outlined in previous research [37], parents may be more likely to register their child if they are having dental pain or problems. Discretion, therefore, should be taken regarding the generalisation of this data sample.

We found no evidence of bias between the baseline characteristics of dyads who did and didn't complete follow-up, thus our interpretation assumes there is no bias between the groups. 'Complete' follow-up was defined as dyads who participated in all three home visits and fully completed plaque scores—i.e. the dyads most compliant with the research—to ensure no biases were overlooked.

Collecting dietary data presented several challenges. The dietary questionnaire, although a validated data collection method [28], had limitations: it relied on recall, parents were sometimes unsure of diet at nursery/with grandparents, and it did not account for seasonal differences. Anecdotally, researchers found some parents struggled with definitions used in the frequency table (e.g. ‘sugar-free' as opposed to ‘no added sugar’ beverages). Revision of the dietary data collection method will be considered prior to progression to full trial. Specific high-risk dietary behaviours—such as the consumption of sugar immediately before bedtime—can be reported more reliably and has been significantly related to caries experience [46].

For observed measures of PSB, such as plaque score and toothbrushing duration, presence of the research team could have induced an observer effect (parents brushing for longer when being filmed). It was planned to assess toothbrushing frequency using the Disney Magic Timer smartphone application, however, there was mixed uptake of this method that yielded little useable data. There are also limitations to the extent of which the application can be considered an objective data collection method – the toothbrush is not synced to the application therefore it could be activated when no toothbrushing is occurring. Barriers for use are discussed in the qualitative paper from this study [26].

## Conclusion

This early phase study has shown that "Strong Teeth" is a feasible oral health intervention for dental practices and parents of children aged 0–5 years old. Data collection in the home setting was acceptable to those families recruited and provided a valuable insight into home-life, including the filming of toothbrushing behaviours in the home setting. The quantitative data has shown a positive indication of impact on compliance to national toothbrushing guidelines and a reduction in plaque levels of children aged 3–5 who used the electric toothbrush. Reported dietary habits remained unchanged. The recruitment and retention of children aged 0–2 was challenging and, if this younger cohort of children was to be included, amendments to the study design would be required before progression to full trial.

## Supplementary Information

**Additional file 1**. “Strong Teeth” Self-Report Questionnaire.

## Data Availability

"Strong Teeth" [49] resources and the protocol paper [50] is available online. Further information is available from the author on reasonable requests.

## Abbreviations

BL: Baseline
BASCD: British association of the study of community dentistry
DBOH: Delivering better oral health
DenTCRU: Dental translational and clinical research unit
dmft: Decayed missing and filled teeth (primary teeth)
IMD: Index of multiple deprivation
MRC: Medical research council
NICE: National Institute of Clinical Excellence
NIHR: National Institute of Health Research
NHS: National Health Service
PSB: Parental-supervised brushing
RCT: Randomised control trial
2MFU: 2-Month follow-up
2WFU: 2-Week follow-up
